# Vitamin K2 as a New Modulator of the Ceramide De Novo Synthesis Pathway

**DOI:** 10.3390/molecules26113377

**Published:** 2021-06-03

**Authors:** Adrian Kołakowski, Piotr F. Kurzyna, Hubert Żywno, Wiktor Bzdęga, Ewa Harasim-Symbor, Adrian Chabowski, Karolina Konstantynowicz-Nowicka

**Affiliations:** Department of Physiology, Medical University of Bialystok, 15-089 Bialystok, Poland; adriankolakowski17@gmail.com (A.K.); pf.kurzyna@gmail.com (P.F.K.); hubert.zywno@gmail.com (H.Ż.); wbzdega@gmail.com (W.B.); eharasim@umb.edu.pl (E.H.-S.); adrian@umb.edu.pl (A.C.)

**Keywords:** sphingolipids, vitamin K2, ceramide, insulin resistance, ceramide, hepatocytes

## Abstract

The aim of the study was to evaluate the influence of vitamin K2 (VK2) supplementation on the sphingolipid metabolism pathway in palmitate-induced insulin resistant hepatocytes. The study was carried out on human hepatocellular carcinoma cells (HepG2) incubated with VK2 and/or palmitic acid (PA). The concentrations of sphingolipids were measured by high-performance liquid chromatography. The expression of enzymes from the sphingolipid pathway was assessed by Western blotting. The same technique was used in order to determine changes in the expression of the proteins from the insulin signaling pathway in the cells. Simultaneous incubation of HepG2 cells with palmitate and VK2 elevated accumulation of sphinganine and ceramide with increased expression of enzymes from the ceramide de novo synthesis pathway. HepG2 treatment with palmitate and VK2 significantly decreased the insulin-stimulated expression ratio of insulin signaling proteins. Moreover, we observed that the presence of PA w VK2 increased fatty acid transport protein 2 expression. Our study showed that VK2 activated the ceramide de novo synthesis pathway, which was confirmed by the increase in enzymes expression. VK2 also intensified fatty acid uptake, ensuring substrates for sphingolipid synthesis through the de novo pathway. Furthermore, increased concentration of sphingolipids, mainly sphinganine, inhibited insulin pathway proteins phosphorylation, increasing insulin resistance development.

## 1. Introduction

Sphingolipids are a class of lipids that, among other functions, are also the structural components of cellular membranes. Because of their enigmatic nature they were named after a mythological sphinx after their discovery in the 1870s in brain extracts. Sphingolipids such as ceramide (CER), sphinganine (SFA) and sphingosine (SFO) are signaling molecules that modulate cell-to-cell interactions and regulate cell differentiation and proliferation. What is more, they may have a significant impact on the insulin signaling pathway, especially during their increased accumulation in hepatocytes caused by excessive free fatty acids (FFA) influx to the liver. The sphingolipid metabolism pathway is an important cellular pathway containing a system of linked reactions in which ceramide occupies a central place. It can be generated through de novo synthesis, hydrolysis of sphingomyelin and the salvage pathway. However, most important in the pathogenesis of insulin resistance is the de novo pathway, which begins in the endoplasmic reticulum by the action of the enzyme serine palmitoyltransferase 1 and 2 (SPTLC1 and SPTLC 2), producing 3-ketosphinganine from serine and palmitoyl-CoA. 3-ketosphinganine after reduction to sphinganine forms dihydroceramide, a process catalyzed by dihydroceramide synthases (LASS4 and LASS6), which are enzymes that share two pathways: de novo and salvage. As a result of this pathway activity, ceramide is synthetized. It can also be formed in the salvage pathway from free sphingosine under the influence of dihydroceramide synthase 4 and dihydroceramide synthase 6. Formed ceramide may also undergo the catabolic pathway with the action of acid (ASAH1), neutral (ASAH2) and alkaline ceramidase (ASAH3), releasing sphingosine as a result [1,2,3].

Insulin resistance (IR) is a condition that can be described as a lack of insulin effects on selected tissues, mostly due to impaired post-receptor signaling pathways [4]. There are studies showing that insulin resistance development is a consequence of the elevated concentration of ceramide in the cell, which may influence the insulin signaling pathway by distinct mechanisms that are not entirely clear. However, inhibition of insulin receptor substrate 1 (IRS-1) and protein kinase B (Akt) phosphorylation by excessive ceramide accumulation seem to be the potential sites of ceramide impairment in this pathway [5]. The repercussions of insulin resistance lead not only to carbohydrate metabolism disorders but also to lipid metabolism disorders and excessive activation of inflammation. Due to those impairments, people with insulin resistance often suffer from non-alcoholic fatty liver disease, type 2 diabetes mellitus and atherosclerosis, which are the most common diseases in humans [6].

Vitamin K2 (VK2, menaquinone) is one of the two most often found types of vitamin K in the human diet and is also synthesized by gut bacteria conversion of vitamin K1 (VK1). Vitamin K2 has several subtypes, determined by izoprene chain length, among which the most important and most commonly described are menaquinone-4 and menaquinone-7 [7,8]. The therapeutic effect of vitamin K2 has been described in the treatment of patients with liver cirrhosis and was tested as a potentially protective drug in type 2 diabetes mellitus [7,9]. In the present study we sought to outline the beneficial effect of menaquinone on insulin resistance development in order to better understand the interplay between vitamin K2 supplementation and accumulation of sphingolipids that interfere with the insulin signaling pathway.

## 2. Results

### 2.1. Effects of HepG2 Cell Incubation with PA and/or VK2 on Sphingolipid Concentration

**Figure 1:**
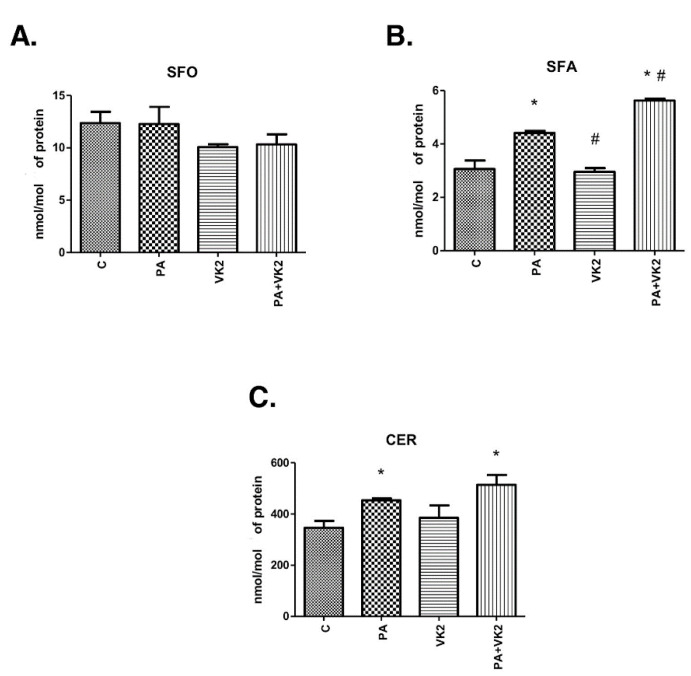
Intracellular concentration of sphingosine (SFO) (**A**), sphinganine (SFA) (**B**) and ceramide (CER) (**C**) in HepG2 cells. The cells were incubated for 16 h with palmitic acid and/or vitamin K2. The sphingolipids concentration was measured by high-performance liquid chromatography as described in the Materials and Methods section. The results are presented as mean ± SD and are based on six independent determinations. *—*P* < 0.05 significant difference vs control group, #—*P* < 0.05 significant difference vs palmitate group; C—control, PA—palmitate, VK2—vitamin K2, PA + VK2—palmitate and vitamin K2.

In our study we measured sphingolipid (sphingosine, sphinganine and ceramide) concentrations in order to discover whether vitamin K2 influenced ceramide accumulation and what sphingolipid fraction was the most affected. The exposure of human hepatocellular carcinoma cells to palmitic acid alone or in combination with vitamin K2 did not reveal statistically significant changes in sphingosine concentration (Figure 1A). Incubation of HepG2 cells with PA alone or with VK2 and PA significantly increased ceramide (PA: +31.1%, PA + VK2: +48.6%, *P* < 0.05) and sphinganine (PA: +44.1%, PA + VK2: +83.6%, *P* < 0.05) accumulation compared with the control group (Figure 1B,C). In addition, we noticed that the usage of VK2 together with PA significantly elevated sphinganine concentration (PA + VK2: +27.4%, *P* < 0.05) compared with PA alone. Furthermore, sphinganine accumulation (VK2: −33%, *P* < 0.05) decreased in VK2 group compared with the PA alone group (Figure 1B). However, simultaneous incubation with PA and VK2 caused a trend towards increase in CER content, compared with the PA alone group, but it did not reach a statistically significant level (*P* = 0.1936) (Figure 1C).

### 2.2. Effects of HepG2 Cells Incubation with PA and/or VK2 on Enzymes from Ceramide De Novo Synthesis and Salvage Pathways

**Figure 2:**
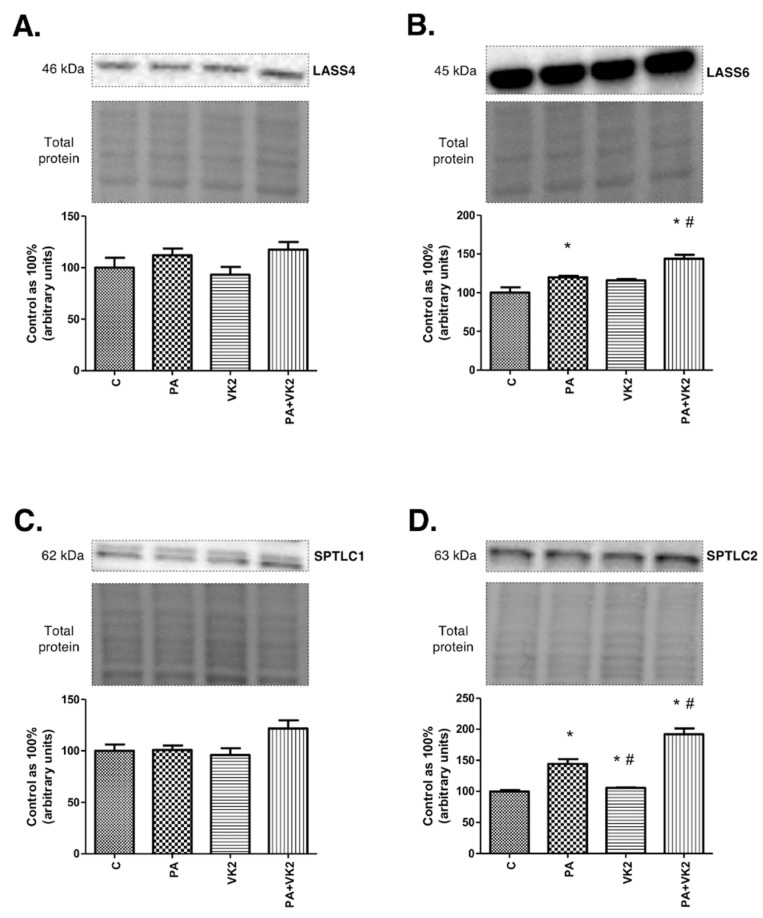
The expression of dihydroceramide synthase 4 (LASS4) (**A**), dihydroceramide synthase 6 (LASS6) (**B**), serine palmitoyltransferase 1 (SPTLC1) (**C**) and serine palmitoyltransferase 2 (SPTLC2) (**D**) in HepG2 cells. The cells were incubated for 16 h with palmitic acid and/or vitamin K2. The expression of enzymes involved in de novo synthesis and salvage pathways were assessed by Western blotting as described in the Materials and Methods section. The results are presented as mean ± SD and are based on six independent determinations. *—*P* < 0.05 significant difference vs control group, #—*P* < 0.05 significant difference vs palmitate group; C—control, PA—palmitate, VK2—vitamin K2, PA + VK2—palmitate and vitamin K2.

The expression of enzymes from de novo synthesis and salvage pathways, two main routes of ceramide formation, was measured in order to establish if those pathways were influenced by vitamin K2. As mentioned previously, dihydroceramide synthases 4 and 6 catalyze formation of dihydroceramide from sphinganine (de novo pathway) and ceramide from sphingosine (salvage pathway), while serine palmitoyltransferase 1 and 2 are responsible for synthesis of ketosphinganine from serine and palmitoyl-CoA. The exposure of HepG2 cells to PA alone or in combination with VK2 did not reveal statistically significant changes in dihydroceramide synthase 4 and serine palmitoyltransferase 1 expression in comparison with the control as well as PA groups (Figure 2A,C). The exposure of HepG2 cells to palmitic acid and/or vitamin K2 caused a considerable increase in LASS6 (PA: +19.9%, PA + VK2: +43.9%, *P* < 0.05) and SPTLC2 (PA: +44.2%, VK2: +5.8%, PA + VK2: 91.9%, *P* < 0.05) expression in comparison with the control group. Incubation with PA combined with VK2 also resulted in a significant increase in LASS6 (PA + VK2: +20.1%, *P* < 0.05) and SPTLC2 (PA + VK2: +33.1%, *P* < 0.05) expression compared with the group incubated with PA alone. Unexpectedly, we also noticed that SPTLC2 expression was significantly decreased in HepG2 cells incubated with vitamin K2 alone compared with the group incubated with palmitic acid (VK2: −26.6%, *P* < 0.05) (Figure 2B,D).

### 2.3. Effects of HepG2 Cells Incubation with PA and/or VK2 on Expression of Enzymes from Ceramide Catabolic Pathway

**Figure 3:**
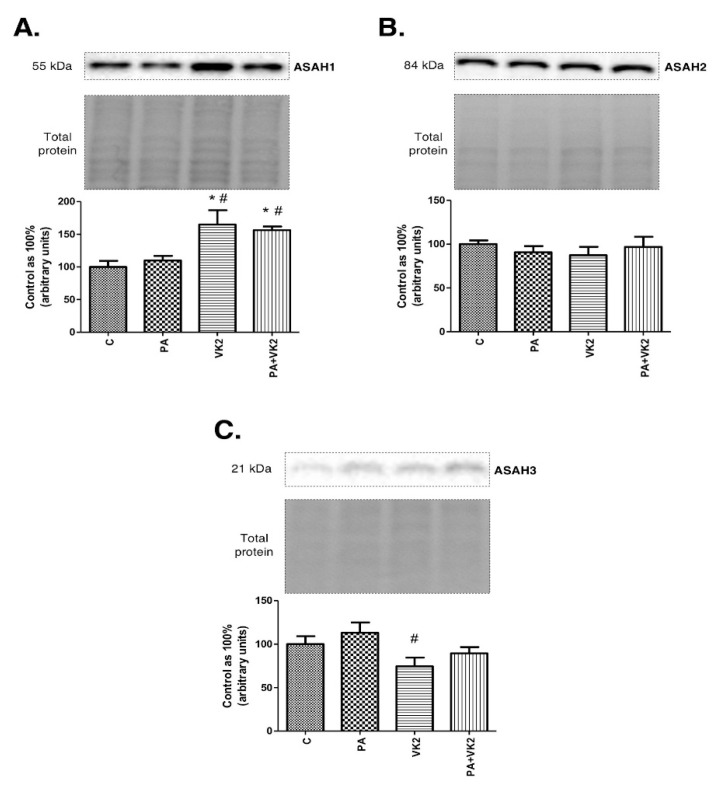
Intracellular expression of acid ceramidase (ASAH1) (**A**), neutral ceramidase (ASAH2) (**B**) and alkaline ceramidase (ASAH3) (**C**) in HepG2 cells. The cells were incubated for 16 h with palmitic acid and/or vitamin K2. The expression of enzymes from ceramide catabolism pathway was assessed by Western blotting as described in the Materials and Methods section. The results are presented as mean ± SD and are based on six independent determinations. *—*P* < 0.05 significant difference vs control group, #—*P* < 0.05 significant difference vs palmitate group; C—control, PA—palmitate, VK2—vitamin K2, PA + VK2—palmitate and vitamin K2.

In order to elucidate if vitamin K2 may enhance ceramide catabolism, we assessed the expression of enzymes responsible for this process—namely, acid, neutral and alkaline ceramidase. Expression of ASAH1 in HepG2 cells incubated with palmitic acid and/or vitamin K2 was significantly increased compared with the control group (VK2: +65.2%, PA + VK2: +56.5%, *P* < 0.05). Moreover, the same groups caused a considerable increase in ASAH1 (VK2: +50.3%, PA + VK2: +42.4%, *P* < 0.05) expression in comparison with the group treated with palmitic acid. (Figure 3A). We did not notice any statistically significant changes in expression of ASAH2 (Figure 3B). The expression of ASAH3 was significantly decreased in the group incubated with vitamin K2 (VK2: −25.6%, *P* < 0.05) compared with the control group (Figure 3C).

### 2.4. Effects of HepG2 Cell Incubation with PA and/or VK2 on Insulin Signalling Pathway Proteins Expression

**Figure 4:**
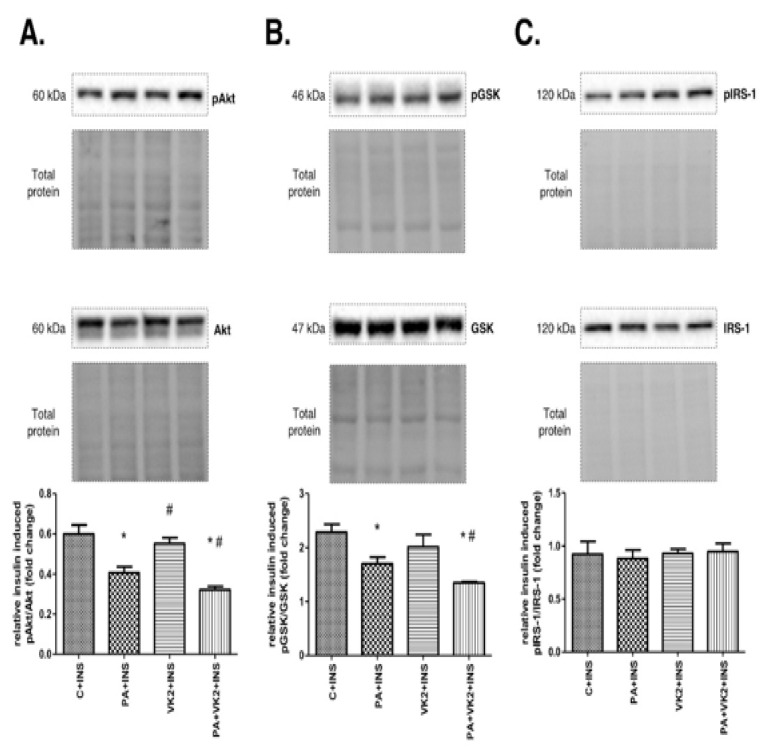
The total expression ratio of phosphorylated and unphosphorylated proteins from insulin signaling pathway—namely, phosphorylated protein kinase B/protein kinase B (pAkt/Akt) (**A**), phosphorylated glycogen synthase kinase/glycogen synthase kinase (pGSK/GSK) (**B**) and phosphorylated insulin receptor substrate 1/insulin receptor substrate 1 (pIRS-1/IRS-1) (**C**). The cells were incubated for 16 h with palmitic acid and/or vitamin K2 with the addition of insulin for last 12 min of incubation. Proteins from insulin signaling pathway were assessed by Western blotting as described in the Materials and Methods section. The results are presented as mean ± SD and are based on six independent determinations. *—*P* < 0.05 significant difference vs control group with addition of insulin, #—*P* < 0.05 significant difference vs palmitate group with addition of insulin; C + INS—control and insulin, PA + INS—palmitate and insulin, VK2 + INS—vitamin K2 and insulin, PA + VK2 + INS—palmitate, vitamin K2 and insulin.

In the next step of our experiment, we wanted to elucidate the influence of vitamin K2 on palmitate-induced insulin resistance. Thus, we assessed the expression of proteins and its phosphorylated forms from the insulin signaling pathway. HepG2 cells incubated with insulin and palmitic acid or with insulin, palmitic acid and vitamin K2 simultaneously expressed decreased pAkt/Akt (PA + INS: 1.48-fold lower, PA + VK2 + INS: 1.86-fold lower, *P* < 0.05) and pGSK/GSK (PA + INS: 1.34-fold lower, PA + VK2 + INS: 1.69-fold lower, *P* < 0.05) ratio compared with the control and insulin group. The exposure of cells to insulin, PA and VK2 simultaneously, significantly reduced the expression ratio of these proteins—namely, pAkt/Akt (PA + VK2 + INS: 1.26-fold lower, *P* < 0.05) and pGSK/GSK (PA + VK2 + INS: 1.26-fold lower, *P* < 0.05) compared with the group incubated with PA and INS. Unexpectedly, we also noticed that the pAkt/Akt ratio was significantly increased in HepG2 cells incubated with vitamin K2 and insulin, compared with the group incubated with palmitic acid and insulin (VK2 + INS: 1.36-fold higher, *P* < 0.05) (Figure 4A,B). We did not reveal statistically significant changes in the pIRS-1/IRS-1 ratio (Figure 4C).

### 2.5. Effects of HepG2 Cells Incubation with PA and/or VK2 on Expression of Proteins Involved in Fatty Acid Transport

**Figure 5:**
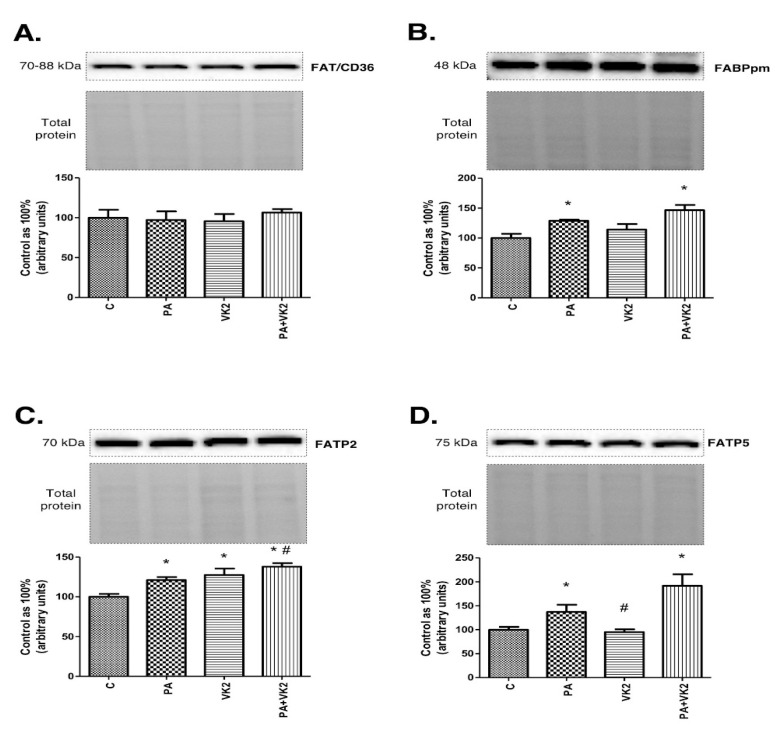
The expression of fatty acid translocase (FAT/CD36) (**A**), fatty acid-binding proteins (FABPpm) (**B**), fatty acid transport proteins 2 (FATP2) (**C**) and fatty acid transport proteins 5 (FATP5) (**D**) in HepG2 cells. The cells were incubated for 16 h with palmitic acid and/or vitamin K2. The expression of proteins involved in plasmalemmal transport of fatty acids were assessed by Western blotting as described in the Materials and Methods section. The results are presented as mean ± SD and are based on six independent determinations. *—*P* < 0.05 significant difference vs control group, #-*P* < 0.05 significant difference vs palmitate group; C—control, PA—palmitate, VK2—vitamin K2, PA + VK2—palmitate and vitamin K2.

To better understand the influence of vitamin K2 on accumulation of sphingolipids, we measured the expression of proteins involved in fatty acid transport to indicate whether and which specific fatty acid transporter could have an impact on palmitate uptake in increased availability of lipids in the media. The exposure of HepG2 cells to PA alone or in combination with VK2 did not reveal statistically significant changes in FAT/CD36 expression (Figure 5A). The presence of PA and PA with vitamin K2 in HepG2 cells caused a substantial increase in FABPpm (PA: +28.9%, PA + VK2: +45.6%, *P* < 0.05) expression compared with the control group (Figure 5B). The expression of FATP2 was significantly increased in all the treated groups (PA: +21.1%, VK2: +27.4%, PA + VK2: +37.9%, *P* < 0.05) in comparison with the control group. Moreover, we noticed relevant growth of FATP2 expression in the group treated with palmitic acid and vitamin K2 simultaneously compared with the group incubated with palmitic acid (PA + VK2: +13.9%, *P* < 0.05) (Figure 5C). The presence of PA and PA with vitamin K2 in HepG2 cells also caused a significant increase in FATP5 expression compared with the control group (PA: +37.2%, PA + VK2: +91.8%, *P* < 0.05) (Figure 5D).

## 3. Discussion

In recent years, one of the main research directions has been the study of the insulin signaling pathway and substances that may affect it. Insulin resistance accompanies many metabolic disorders, such as type 2 diabetes or metabolic syndrome [10]. The insulin signaling pathway is influenced by many genetic and environmental factors, among which one of the main is an increase in plasma fatty acids concentration, as evidenced by numerous scientific studies [11,12]. In recent years, there has been growing interest in the influence of vitamin K1 and K2 on the insulin signaling pathway and glucose metabolism, especially in the liver. Previous years’ research has shown that vitamin K2 has a beneficial role in improving insulin sensitivity in skeletal muscles [8,13]. However, there are not many studies describing the effects of vitamin K2 on the sphingolipid metabolism pathway in hepatocytes. Therefore, in our research we focused on the influence of vitamin K2 on insulin resistance in hepatocellular carcinoma cells by influencing the sphingolipid pathway. In our research conducted on a human hepatocellular carcinoma cells cell line, we used a palmitic acid that increased the transport of this fatty acid. Incubation of vitamin K2 with palmitic acid significantly increased the expression of fatty acid transport protein 2 compared with the control as well as with the palmitic acid groups. Increased expression of fatty acid transporters led to increased intracellular transport and served the substrates needed for the ceramide de novo synthesis pathway, which is in accordance with studies by Matsumoto et al. in skeletal muscles [13]. It is well known that ceramide is a central component of the sphingolipid pathway and a precursor for many other sphingolipids, e.g., sphingosine or sphingomyelin [1]. Enhanced accumulation of ceramide promotes dephosphorylation of Akt by protein phosphatase 2A (PP2A) or directly inhibits insulin-stimulated phosphorylation of protein kinase B. As a result, there is decreased insulin sensitivity and decreased transport of glucose into the cell [14,15]. In our work, human hepatocellular carcinoma cells incubated with palmitic acid exerted increased concentration of ceramide and developed insulin resistance. The direct influence of vitamin K2 incubated with palmitic acid on human hepatocellular carcinoma cells resulted in an increased accumulation of sphinganine compared with the use of palmitic acid alone. However, simultaneous incubation with palmitic acid and vitamin K2 caused a trend towards increase in ceramide content but it did not reach a statistically significant level. We may suspect that vitamin K2 stimulated the activity of the de novo sphingolipids synthesis in both human hepatocellular carcinoma cells treated only with vitamin K2 and those characterized with insulin resistance. The confirmation of the above results is the increase in the expression of the two enzymes participating in the ceramide de novo synthesis pathway—serine palmitoyltransferase 2 and dihydroceramide synthase 6, the expression of which increased relative to the group with palmitic acid. There are studies confirming that vitamin K2 may increase sphingolipids concentration. One of them was conducted by Denisova et al., who showed that vitamin K2 deficiency lowered the concentration of sphingolipids in the mouse brain. In the same work, scientists described the stimulating effect of vitamin K1 and vitamin K2 on the enzyme galactosyl-ceramide sulfate transferase activity, which is involved in the synthesis of complex sphingolipids, such as sulfatides [16,17]. Based on results conducted on the brain and those shown in our study on steatotic human hepatocellular carcinoma cells, we may suspect that vitamin K2 can act on various tissues differently and influence the accumulation of other compounds from sphingolipid family through other synthetic routes than those described in our work. This shows that much research is still needed to elucidate the effects of vitamin K2 on the individual tissues. Lev et al. also described the effect of vitamin K1 on the activity of serine palmitoyltransferase but in Bacteroides melaninogenicus or Fusiformis nigrescens [18,19]. Over time, research on bacteria was extended to the use of vitamin K2 in rodent models and confirmed the previously obtained results [20,21]. Thus, vitamin K1 and vitamin K2 significantly accelerated the enzyme expression that induced de novo synthesis and led to the formation of 3-Ketosphinganine, which is the first sphingolipid in the ceramide synthesis pathway [20]. This is in line with our results in which we showed increased expression of serine palmitoyltransferase 2 and dihydroceramide synthase 6 leading to increased ceramide synthesis. However, it should be remembered that some of research was conducted on bacteria, with the addition of vitamin K1 [22]. It means that the effect observed in bacteria cannot be directly extrapolated to human cells and that these two isoforms, vitamin K1 and K2, may react differently. Considering other components from the sphingolipid metabolism routes, the lack of vitamin K2 effect on the concentration of sphingosine in all the studied groups was noticeable, which proves that a lack of vitamin K2 has influence on the sphingolipid salvage pathway. Moreover, we observed no significant changes compared with the palmitic acid group in the expression of neutral and alkaline ceramidases, which are enzymes involved in the formation of sphingosine via this pathway. It should be noted that the concentration of acid ceramidase increased significantly. However, the expression of this enzyme in the liver is on trace level, which greatly reduces the importance of acid ceramidase [23]. It is well known that sphingolipids, such as ceramide, inhibit the insulin signaling pathway. Our results showed the inhibitory effect of palmitic acid incubation, especially with vitamin K2, on insulin-stimulated phosphorylation of protein kinase B and glycogen synthase kinase (GSK) compared with the group incubated with palmitic acid and insulin alone. It proved the additive effect of vitamin K2 on cells characterized with insulin resistance and led to the deterioration of this disorder. In contrast, there are some studies describing the ameliorating effects of vitamin K2 on insulin resistance development. Xiangni et al. have shown that naturally occurring vitamin K2 reduced free fatty acids accumulation, increased mitochondrial biogenesis through the SIRT1 signaling pathway and led to increased glucose tolerance in skeletal muscles [8]. We can explain the differences between our study and those cited above by the different experimental models. Our studies were conducted on human derived cells, which could react differently from the skeletal muscles that were derived from animals. Choi et al., described that vitamin K2 decreased whole body insulin resistance by influencing osteocalcin metabolism. They suggested that human vitamin K2 supplementation for 4 weeks led to an increase in carboxylated osteocalcin (cOC), which increased the sensitivity of the whole body to insulin [24]. It should be remembered that our research was conducted on liver cells whose insulin resistance was induced by palmitate incubation compared to studies of Choi et al., who examined vitamin K2 effect on insulin sensitivity of young healthy volunteers. However, the data elucidating the precise mechanism of vitamin K2 effects on the insulin pathway were not described. Neither of them focused on the activity of protein kinase B, the activity of which may be modulated by changes in the concentration of sphingolipids. Holland et al. described the lipotoxic effect of ceramide on insulin action by inhibiting protein kinase B phosphorylation by activating protein phosphatase 2A and the dephosphorylation of protein kinase B or by blocking translocation of protein kinase B to the plasma membrane [25]. Furthermore, Chaurasia et al. reported that pharmacological inhibition of sphingolipid synthesis prevented insulin resistance development caused by high fat feeding in rodent models [26]. In our studies, the expression of the insulin receptor substrate 1 did not change significantly, which means that vitamin K2 acted directly on downstream proteins from the insulin signaling pathway. However, more studies are still needed to confirm our results, especially in nonmalignant human liver cells.

## 4. Materials and Methods

### 4.1. Cell Culture

All the experiments were performed on human hepatocellular carcinoma cells (Hep G2/C3A) purchased from the American Type Culture Collection (ATCC, Manassas, VA, USA). Human hepatocellular carcinoma cells were maintained in high-glucose (4.5 g/L) Dulbecco’s modified Eagle’s medium (DMEM, PAN-Biotech, Aidenbach, Germany) containing 10% Fetal Bovine Serum (FBS, BioWest, Nuaillé, France) and 1% antibiotic/antimycotic (penicillin-streptomycin, PAN-Biotech, Aidenbach, Germany). The cells were cultured for 7 days in a 5% CO_2_ humidified atmosphere at 37 °C up to the 70% confluence. The medium was replaced every 48 h. After 7 days the cells were seeded in 6-well plates and cultured in standard growth medium to achieve 90% of confluency. The morphology and viability of the attached cells, after twice washing with PBS (PAN-Biotech, Aidenbach, Germany), was assessed in Bürker chamber using Trypan blue (Sigma-Aldrich, St. Louis, MO, USA) staining. The experiment was carried out on plates where the percentage of living cells was above 85%.

### 4.2. Experimental Procedure

The treatment with palmitic acid (PA) was conducted on one-hour serum-starved cells. At first, palmitate was dissolved in a mixture of ethanol and 1 M NaOH, heated to 70 °C, then conjugated with 10% fatty acid-free bovine serum albumin (BSA, Sigma-Aldrich, St. Louis, MO, USA) and diluted in DMEM as it was described previously [8]. The HepG2 cells were divided into four experimental groups, which were incubated for 16 h at 37 °C in 5% CO_2_ atmosphere. The first group was a control group incubated in medium supplemented with 10% BSA. In the second group, hepatocytes were incubated with palmitic acid (0.5 mM) in order to induce insulin resistance. The third group was incubated for 16 h in the same medium as the control group with the addition of 30 μM vitamin K2 (VK2, Sigma-Aldrich, St. Louis, MO, USA), while the fourth group was treated with palmitic acid combined with vitamin K2. The concentrations of vitamin K2 used in our experiment were selected based on preliminary studies and publications where 30 µM of vitamin K2 was used [27,28]. Furthermore, in the second set of experiments, the same four experimental groups were treated with the addition of 100 nM insulin (NOVORAPID, Bagsvaerd, Denmark) for last 12 min of 16 h incubation. At the end of the experiment, the cells were washed three times with ice-cold PBS, homogenized in ice-cold RIPA lysis buffer containing protease and phosphatase inhibitors (Roche Diagnostics GmbH, Mannheim, Germany), ultrasonicated and frozen.

### 4.3. Immunoblotting Analyses

The expression of proteins—protein kinase B (Akt, 1:500, Cell Signaling Technology, Danvers, MA, USA), glycogen synthase kinase (GSK, 1:500, Invitrogen, Rockford, IL, USA), insulin receptor substrate 1 (IRS-1, 1:1000, Cell Signaling Technology, Danvers, MA, USA) and their phosphorylated forms phosphorylated protein kinase B (pAkt, 1:1000, Cell Signaling Technology, Danvers, MA, USA), phosphorylated glycogen synthase kinase (pGSK, 1:500, Invitrogen, Rockford, IL, USA) and phosphorylated insulin receptor substrate 1 (pIRS-1, 1:1000, Cell Signaling Technology, Danvers, MA, USA) which are directly involved in the insulin signaling pathway, as well as the sphingolipid pathway enzymes namely, dihydroceramide synthase 4 (LASS4, 1:500, Santa Cruz Biotechnology, Inc., Dallas, TX, USA), dihydroceramide synthase 6 (LASS6, 1:500, Abcam, Cambridge, UK), serine palmitoyltransferase 1 (SPTLC1, 1:500, Abcam, Cambridge, UK), serine palmitoyltransferase 2 (SPTLC2, 1:500, Santa Cruz Biotechnology, Inc., Dallas, TX, USA), acid ceramidase (ASAH1, 1:500, Santa Cruz Biotechnology, Inc., Dallas, TX, USA), neutral ceramidase (ASAH2, 1:200, Santa Cruz Biotechnology, Inc., Dallas, TX, USA) and alkaline ceramidase (ASAH3, 1:500, Novus Biologicals, Abingdon, UK) were detected by Western blot procedure. Moreover, the expression of fatty acid transporters—fatty acid translocase (FAT/CD36, 1:500, Santa Cruz Biotechnology, Inc., Dallas, TX, USA), fatty acid-binding protein (FABPpm, 1:8000, Santa Cruz Biotechnology, Inc., Dallas, TX, USA), fatty acid transport proteins 2 (FATP2, 1:200, Santa Cruz Biotechnology, Inc., Dallas, TX, USA) and 5 (FATP5, 1:500, Abcam, Cambridge, UK)—were measured using this technique, as was described previously by Konstantynowicz-Nowicka et al. [5]. In brief, before the Western blot procedure, total protein concentration was assessed with the bicinchioninic acid method, using BSA as a standard. Cell lysate probes (30 µg of protein per line) were separated by 10% Criterion TGX Stain—Free Precast Gel (Bio Rad, Warsaw, Poland) electrophoresis and transferred onto nitrocellulose membranes. After blocking with 5% nonfat dry milk or 5% BSA for phosphorylated proteins, the membranes were immunoblotted with primary antibodies of interest and then incubated in 4 °C overnight. Afterwards, the blots were incubated with appropriate horseradish peroxidase—labeled secondary antibodies. The ChemiDoc EQ (Bio Rad, Warsaw, Poland) visualization system was used to densitometrically quantify received signals. Ponceau S (Sigma-Aldrich, St. Louis, MO, USA) staining was used in order to confirm equal protein loading. The expression of each analyzed protein was standardized to the total protein expression, and the control group was set as 100%.

### 4.4. Intracellular Sphingolipid Snalyses

The ceramide, sphinganine and sphingosine concentrations were estimated by high-performance liquid chromatography (HPLC) according to the method developed by Min et al. [29], which was widely described in Konstantynowicz-Nowicka et al. [5]. Briefly, lipids from sonicated hepatocytes were extracted in the presence of internal standard—namely, N-palmitoyl-D-erythro-sphingosine, C17 base. Then aliquots of the lipid extracts were transferred to a fresh tube with pre-added 40 pmol of internal standard (N-palmitoyl-D-erythro-sphingosine) and next subjected to alkaline hydrolysis to form deacylate ceramide. Free sphinogosine and sphinganine—two metabolites released from ceramide during that process—were then converted to their o-phthalaldehyde derivatives and analyzed by the HPLC system (PROSTAR; Varian Inc., Palo Alto, CA, USA) equipped with a fluorescence detector and C18 reversed-phase column (Varian Inc., Palo Alto, CA, USA, OmniSpher 5, 4.6 × 150 mm). Estimated sphingolipids’ concentration was expressed in nanomoles per protein concentration in particular sample.

### 4.5. Statisical Analysis

The data are expressed as mean values ± SD based on six independent determinations. The normality of the data distribution and homogeneity of the variance were checked with the use of Shapiro–Wilk test and Bartlett’s test, respectively. The statistical analysis of the results was performed in GraphPad Prism 5 software (La Jolla, CA, USA) with the use of two-way ANOVA followed by an appropriate post hoc test (pairwise Student’s *t*-test). The results were considered as statistically significant at the *P* < 0.05 level.

## 5. Conclusions

In summary, our study demonstrates that vitamin K2 may affect sphingolipid concentration in human hepatocellular carcinoma cells. The main change showed in our study was the increase in sphinganine accumulation after palmitate treatment that was enhanced by vitamin K2. Observed changes were the effect of ceramide de novo synthesis pathway activation, which was confirmed by the increase in enzymes expression. What is more, vitamin K2 also intensified fatty acid transport, ensuring substrates for sphingolipid synthesis through the de novo pathway. Furthermore, increased concentration of sphingolipids affected the insulin signaling pathway, causing inactivation of protein kinase B and glycogen synthase kinase, which increased insulin resistance development. It is also worth mentioning that our study may have clinical significance in obese people with coagulation disorders or osteoporosis who are treated with vitamin K2. These individuals may be characterized by a high availability of fatty acids in the plasma and hypercholesterolemia, which indicates a significant predisposition to the development of diabetes or pre-diabetes. As a result, vitamin K2 supplementation in people with these chronic diseases may induce the development of insulin resistance, so special attention should be paid to these groups of patients. However, additional clinical trials are required to support the above conclusion, and our research is just a prelude to further research. The cell line used in our study has limitations in drawing clear conclusions, while also being one of the more reliable since it exerts many of the metabolic properties of normal human hepatocytes. Testing more cell lines will increase the reliability of the obtained results, which means that in the near future we intend to conduct research on other types of cells, such as human hepatocyte cell lines HeparRG and IHH as well as primary rat hepatocytes.

## Data Availability

The data presented in this study are available on request from the corresponding author. The data are not publicly available due to Medical University of Białystok policy.

## Sample Availability

Samples of the synthetic vitamin K2 is commercially available in Sigma Aldrich and cell culture is available from the authors.
